# Supplementary material for: The awareness of public about the Emergency Medical Services in the Eastern region of Saudi Arabia
Source: PLoS One. 2024 Jul 11;19(7):e0306878. doi: 10.1371/journal.pone.0306878 (PMC11238984; doi:10.1371/journal.pone.0306878)
Supplement: Attachment — Submitted filename: Response to Reviewers.docx [file pone.0306878.s002.docx]

Dear Dr. Alfuqaha,

I am writing to submit the revised version of our manuscript, titled The Awareness of Public about the Emergency Medical Services in the Eastern Region of Saudi Arabia, for consideration in PLOS ONE. Following your valuable feedback and suggestions, we have made several enhancements to our manuscript.

We have meticulously addressed each point raised by the reviewers and have included a rebuttal letter outlining our responses to their insightful comments. Furthermore, we have revised our manuscript to include more detailed information on participant consent and data collection methods, as well as enhanced the clarity of our figures and tables.

We believe these revisions have significantly improved the quality of our manuscript and align it more closely with PLOS ONE's publication criteria. The revised manuscript, along with a marked-up copy highlighting the changes and an unmarked version, is attached for your review.

We appreciate the opportunity to revise our work and hope that it now meets the standards for publication in PLOS ONE. We look forward to your feedback and are happy to provide any further information or make additional revisions if required.

Thank you for considering our manuscript for publication.

Sincerely,

Dr.Ahmed Alanazy

| Comment | Authors Reply |
| --- | --- |
| Journal Requirements: | |
| 1) Please ensure that your manuscript meets PLOS ONE's style requirements, including those for file naming. | The manuscript was edited according to the provided model |
| 2) Please provide additional details regarding participant consent. In the ethics statement in the Methods and online submission information, please ensure that you have specified (1) whether consent was informed and (2) what type you obtained (for instance, written or verbal, and if verbal, how it was documented and witnessed). If your study included minors, state whether you obtained consent from parents or guardians. If the need for consent was waived by the ethics committee, please include this information. | The consent details were added |
| 3) Please include a caption for Figure 2. | Caption was added |
| 4) Please include a copy of Table 3 which you refer to in your text on page 8 of 13. | Table was added |
| 5) We note you have included a table to which you do not refer in the text of your manuscript. Please ensure that you refer to Table 4 in your text; if accepted, production will need this reference to link the reader to the Table. | The table number was edited |
| Editor Comments: | |
| 1) Title: Shorten the title to no more than 15 words for conciseness. | The title was shorten |
| Abstract: Include details about your data collection methods such as whether you used random, snowball, or convenience sampling. | The sampling technique was added to the abstract and in the method section |
| Introduction: Clarify whether the deficiency in public awareness of EMS is specific to Eastern Saudi Arabia, the entire Kingdom of Saudi Arabia, or if it extends globally. Broaden the scope in the introduction.  Introduce a paragraph outlining the unique contributions of your study and compare them with findings from previous research. | The introduction has been updated |
| Method Section: Provide more information on how you conducted the inline survey, ensuring it is clear how participants from the Eastern region or other countries were able to respond. | Added |
| Figures: Consolidate figures to present gender and educational levels in a single chart for clarity. | our decision to present educational levels with gender distribution aligns directly with our study's objective to assess gender differences in EMS awareness. This approach is crucial to our analysis, providing a nuanced understanding of the interplay between gender and education in the context of EMS knowledge. |
| Typos: Carefully review and correct any typos present in the manuscript. | The manuscript was checked |
| Discussion: Begin the discussion section with a focus on your main findings rather than repeating the study's aims. International Comparisons: Compare your results with findings from international studies to highlight key differences. | the dissection has been updated |
| References: Double-check and ensure the accuracy of your references. | Checked |
| Reviewer #1 comments | |
| 1) Underline number 60 and kindly change from “aged between 18 and 60” to “aged between 18 and 60 years | The word “years” was added |
| 2) Kindly add exclusion criteria | The exclusion criteria were added |
| 3) How consent was taken for participation in the survey | The consent details were added |
| 4) Kindly clarify how the survey population were identified for the survey 5) How selection bias was minimized in the survey | Details regarding the survey population identification were added |
| 6) Please scan your manuscript again and correct some topological errors |  |
| 7) Please make sure your manuscript was written in line with this journal submission guideline | The manuscript was edited according to PLOS ONE criteria |
| Reviewer #2 Comments | |
| 1) Abstract needs editing – for example it would be help full to present the general awareness before presenting the contributing factors such as age, gender, and degree. | The awareness was introduced first. |
| 2) The objective in the introduction presents the study design when authors mentioned “our cross-sectional study …etc.” it would be better to move to the method section. | This sentence was edited accordingly. |
| 3) Power calculation is fine and adequate try to replace the website link by the software used to calculate the sample size. | This site has no related software |
| 4) I do not get the grasp from figure 1 for the gender difference. | The figure number was edited |
| 5) Presenting the psychometric properties for the questioner could be confusing. Need more clarification for example how did authors measure reliability (time frame – usually we used 2 weeks intervals between each administration process). I suggest remove the reliability if the questionnaire is valid in measuring what is intended to measure. | We conducted the reliability test to enforce our study. |
| 6) I suggest Knowledge based analysis must be presented first. Then, other factors that can explain the awareness. | The awareness was introduced first. |
| 7) In the logistic regression I would recommend focusing on the adjusted model. Although, the intercepts in tables must be clear in a separate raw. | The we prefer to keep both models to provide a comprehensive overview. The intercepts in tables are already in separate cells. |
| 8) Sort the repetition in the manuscript. for example, the aim of the study was introduced gain in the discussion. | The manuscript was reviewed and repetition was removed. |
| Reviewer #3 Comments | |
| 2) How the author will compare between male and female participants about "Emergency Number 112 Awareness" although P value is 0.37 (nonsignificant), Actions when encountering EMS or EMT (P value= 0.62) and Role of a Dispatcher (P value= 0.56) | While the p-values were non-significant, our study's findings contribute to the broader understanding of emergency response awareness and behavior, suggesting the need for further exploration of gender-related factors. |
| 3) Author mentioned Figures 1 and Figure 2 but there is no "Figure 2" | The figure number was edited |
